# *Saxifraga stolonifera* inhibits porcine epidemic diarrhea virus infection by disrupting nucleocapsid protein-p53 interaction

**DOI:** 10.3389/fcimb.2025.1615300

**Published:** 2025-07-09

**Authors:** Hongde Lu, Haoyang Liu, Ning Guo, Yu Zhou, Haiyan Lu, Zhiyuan He, Hong Dong

**Affiliations:** ^1^ Beijing Key Laboratory of Traditional Chinese Veterinary Medicine, Beijing University of Agriculture, Beijing, China; ^2^ Beijing Engineering Research Center of Chinese Veterinary Medicine, Beijing University of Agriculture, Beijing, China

**Keywords:** PEDV nucleocapsid protein, *Saxifraga stolonifera*, p53-DREAM signaling pathway, cell cycle, network pharmacology, molecular docking

## Abstract

Porcine epidemic diarrhea (PED) is an acute, highly contagious intestinal disease caused by the porcine epidemic diarrhea virus (PEDV), which has devastating effects on the global swine industry. Currently, no effective therapeutic agents have been identified for treating PEDV infections. *Saxifraga stolonifera* (*S. stolonifera*), valued in traditional Chinese medicine for its anti-inflammatory properties, remains poorly studied regarding its efficacy against PEDV. This study demonstrated the dose-dependent inhibition of PEDV nucleocapsid expression by *S. stolonifera in vitro*. *S. stolonifera* strongly inhibited the expression levels of pro-inflammatory cytokines. Using the network pharmacology, key components such as gallic acid, quercetin, coumarin, and caffeic acid were identified. KEGG pathway enrichment analysis revealed that *S. stolonifera* mainly targeted pathways including p53, MAPK, and TNF to exert anti-PEDV effects. *S. stolonifera* treatment disrupted the interaction of PEDV N protein and p53. It also modulated the p53-DREAM signaling pathway by reducing p53 and p21 protein levels, while enhancing p130 (Ser672) phosphorylation, E2F4, and Cyclin A protein expression levels. Molecular docking revealed stable hydrogen bonding between the seven core components and the PEDV N protein, with quercetin exhibiting the lowest binding energy. Amino acid sequence analysis showed that quercetin and other components share conserved binding sites with the PEDV N protein. These findings underscore the potential of *S. stolonifera* as a natural antiviral agent against PEDV infection.

## 1 Introduction

Porcine epidemic diarrhea (PED) is a common swine disease characterized by watery diarrhea, vomiting, and dehydration, with mortality rates reaching up to 100% in suckling piglets (Pensaert and de Bouck, 1978). The porcine epidemic diarrhea virus (PEDV) was first identified in the United Kingdom in 1971. The classical strain CV777, isolated in Belgium in 1978, rapidly spread across Europe, Africa, and Asia, posing a significant threat to the pig-breeding industry (Callebaut et al., 1982; Chen et al., 2008; Puranaveja et al., 2009). Since 2010, a highly virulent mutant strain has emerged in China, causing substantial economic losses. The genomic variability of PEDV strains has rendered existing vaccines ineffective, with pathogenic strains causing mortality rates exceeding 80% in infected piglets (Sun et al., 2012). PEDV is an enveloped, single-stranded, positive-sense RNA virus, classified under the order Nidovirales, family Coronaviridae, and genus Alphacoronavirus. Global PEDV strains are categorized into classical (GI subtype) and variant strains (GII subtype) based on their S gene sequences. These classifications reflect differences in molecular features, antigenic properties, and pathogenic potential (Jung and Saif, 2015). Vaccination remains the primary strategy for preventing and controlling PEDV in clinical practice. The domestic market provides various commercial vaccines, primarily inactivated and live-attenuated vaccines. Inactivated vaccines, however, have limited immunogenicity, requiring multiple booster shots for adequate protection and providing only short-term immunity. Live-attenuated vaccines may undergo reversion to virulence. In addition, coronaviruses, including PEDV, are prone to mutations, facilitating immune evasion and potentially undermining vaccine effectiveness, posing ongoing challenges for disease control (Gerdts and Zakhartchouk, 2017). Therefore, it is urgent to identify and develop antiviral drugs capable of effectively combating PEDV infections.

Antiviral drugs can be classified into three main groups: natural bioactive compounds, herbal extracts, and other bioactive substances. Researchers worldwide are increasingly interested in exploring natural antiviral agents, particularly those derived from the rich tradition of traditional Chinese medicine. Natural compounds, such as flavonoids, polysaccharides, and alkaloids, significantly inhibit PEDV by targeting viral adhesion and internalization and interfering with non-structural proteins, including 3C-like protease (3CLpro), papain-like protease (PLpro), and RNA-dependent RNA polymerase (RdRp), to disrupt viral replication (Chu et al., 2018; Li et al., 2020; Xu et al., 2024). These compounds also alleviate the inflammatory response and regulate host factors (Wang et al., 2024). Previous studies have demonstrated that a blend of heat-clearing herbs enhanced daily weight gain, reduced intestinal villous atrophy and crypt hyperplasia in PEDV-infected piglets, and provided effective protection (Kim et al., 2015). In addition to traditional natural compounds and herbal extracts, bioactive substances derived from algae, microorganisms, and animal products have shown potent antiviral activity against PEDV (Phillips et al., 2022). Surfactin, extracted from the fermentation broth of *Bacillus subtilis*, inhibits PEDV replication and prevents the fusion of viral vesicle membranes with cell membranes, thereby blocking viral invasion (Yuan et al., 2018). Melatonin exhibits significant antiviral effects during the early stages of PEDV invasion and replication. Additionally, chitosan, derived from crustaceans, directly inactivates PEDV particles, highlighting its potential as an antiviral agent (Kim et al., 2021; Zhai et al., 2021).

*S. stolonifera*, traditionally valued for its heat-clearing, detoxifying, dampness-resolving, and wind-dispelling properties, has recently attracted scientific attention for its diverse pharmacological activities. Notably, the alcoholic extract of Nigella sativa, a related species in the *Saxifragaceae*, has exhibited significant antiviral efficacy against RNA viruses, such as HIV-1, in both *in vivo* and *in vitro* studies. Furthermore, the ethyl acetate fraction of this extract inhibits hepatitis C virus (HCV) replication by reducing the activity of the NS3 serine protease. Additionally, this fraction contains polyphenolic monomers with potent antiviral properties (Zuo et al., 2005). Recent studies have underscored the remarkable antiviral potential of *Saxifraga* sp*inulosa*. Its aqueous extract binds directly to SARS-CoV-2, neutralizing 99.6% of the virus by targeting the viral genome and proteome (Takeda et al., 2020).

This study aimed to assess the *in vitro* antiviral effects of *S. stolonifera* against PEDV, focusing on its inhibitory effects and the mechanisms through which it disrupts viral replication. This research seeks to establish a scientific basis for developing novel therapeutic strategies against PEDV and to explore innovative uses of traditional Chinese medicine in preventing and treating PED.

## 2 Materials and methods

### 2.1 Cell cultures

Vero E6 and IPEC-J2 cells were maintained in our laboratory and cultured in Dulbecco’s modified Eagle’s medium (DMEM; Gibco, Grand Island, NY, USA) supplemented with 10% fetal bovine serum (FBS; Gibco, Grand Island, NY, USA) under a humidified atmosphere of 5% CO_2_ at 37°C.

### 2.2 Antibodies and herbs

GAPDH Polyclonal antibody (10494-1-AP), p107 (13354-1-AP), and E2F4 (10923-1-AP) were obtained from Proteintech Group (Wuhan, China). Monoclonal antibodies (MAbs) targeting the PEDV N protein (JN1401) were purchased from JNT (Beijing, China). HRP-labeled Goat Anti-Rabbit IgG (A0208) and HRP-labeled Goat Anti-Mouse IgG (A0216) were purchased from Beyotime (Shanghai, China). Antibodies against p53 (2524) and p21 (2947) were purchased from Cell Signaling Technology (Danvers, MA, USA). Antibodies targeting p130 (ab76234), cyclin A (ab32386), p130 (phospho-S672) (ab76255), and p130 (phospho-S952) (ab68136) were obtained from Abcam (Cambridge, UK). *Saxifrage* hay was obtained from Beijing Tongrentang (Beijing, China).

### 2.3 Virus, virus titration and virus infections

The PEDV strain LW/L was previously isolated and is maintained in our laboratory. Virus was propagated and titrated in Vero E6 cells cultured in DMEM supplemented with 10 μg/mL trypsin (Gibco, Grand Island, NY, USA) under standard incubation conditions. Briefly, 96-well plates were seeded with a monolayer of Vero E6 cells before being infected with serially diluted PEDV (10^–1^ to 10^–10^). The Reed-Muench method was used to calculate the viral titer, expressed as the 50% tissue culture infective dose (TCID_50_), the virus titer of the strain used in this study was calculated to be 10^-6.11^TCID_50_/0.1 mL. For infection assays. Vero E6 cells were infected with the PEDV strain at a multiplicity of infection (MOI) of 0.1.

### 2.4 Preparation of the aqueous extract of *S. stolonifera*

Dried *S. stolonifera* was cut into pieces and soaked in deionized water until fully saturated. The mixture was then brought to a boil, followed by a reduction to a gentle simmer to facilitate the decoction process. After the initial boiling, the liquid was filtered through gauze to separate the plant material. The remaining plant residue was subjected to a second decoction under the same conditions, and the two resulting filtrates were combined. Finally, the combined filtrate was concentrated using a vacuum rotary evaporator to obtain the final extract (Wu et al., 2015).

### 2.5 Cell viability and IC_50_ determination

100 μL of cell suspension was homogeneously inoculated into 96-well plates, mixed and placed in a 5% CO_2_ incubator at 37°C until monolayer; cell control wells and cell-free blank wells were also set up. The prepared *S. stolonifera* was diluted with DMEM medium and filtered using a 0.22 μm filter. The medium was discarded, and different concentrations of *S. stolonifera* were added to the 96-well plate at 100 μL/well, with three replicates for each concentration. After 12 h of drug incubation, the culture solution was discarded, D-Hanks was rinsed twice, and finally 100 μL/well of DMEM culture solution (containing 10% CCK-8 solution) was added, and the incubator was incubated in the light protected for 2 h. The absorbance was measured by an enzyme marker at 450 nm. Half cell inhibition concentration (50% inhibition concentration, IC_50_) was calculated using GraphPad Prism 9.0 software. Cell viability (%) = (OD administration - OD blank)/(OD control - OD blank) × 100%.

### 2.6 Viral replication inhibition assay

We employed five distinct experimental strategies to ascertain the phase of viral inhibition by *S. stolonifera* during PEDV infection. In the all-treatment protocol, cells were exposed to *S. stolonifera* for a duration of 14 h. In the pre-treatment protocol, cells were exposed to *S. stolonifera* for a duration of 1 h prior to the introduction of PEDV. Subsequently, the cells were incubated for an additional 12 h. The co-treatment approach involved the simultaneous inoculation of cells with PEDV, accompanied by *S. stolonifera* treatment for 13 h. The post-treatment methodology commenced with the inoculation of cells with PEDV for 1 h. Post-incubation, the inoculum was aspirated, and the cells were treated with *S. stolonifera* for a subsequent 12 h period. The direct-treatment approach involved the simultaneous inoculation of cells with PEDV, accompanied by *S. stolonifera* treatment for 1 h.

### 2.7 Effects on different stages of virus infection

For virus absorption assessment, cells were seeded in 6-well plates and after pre-cooling at 4°C for 30 min in a refrigerator, the virus maintenance solution was used to infiltrate three times, and a mixture of PEDV LW/L and 200 μg/mL *S. stolonifera* was added and adsorbed for 2 h at 4°C. To evaluate virus internalization, after pre-cooling at 4°C refrigerator for 30 min, the virus maintenance solution was used to infiltrate 3 times, PEDV LW/L was added and incubated at 4°C for 2 h. D-Hank’s was used to infiltrate 3 times, and 200 μg/mL of *S. stolonifera* was added and incubated for 3 h at 37°C. For viral replication assessment, virus maintenance solution was first used to moisten and wash 3 times, inoculated with PEDV LW/L for 1 h. D-Hank’s was used to infiltrate 3 times, and the virus maintenance solution was incubated for 3 h. The virus was discarded, and then added to 200 μg/mL *S. stolonifera* for 12 h of incubation. Viral release was evaluated by using virus maintenance solution to infiltrate 3 times, inoculated with PEDV LW/L for 1 h, washed 3 times with D-Hank’s, incubated with virus maintenance solution for 5 h and discarded, and then added with 200 μg/mL *S. stolonifera* and incubated up to 8 h. Negative and positive control groups were set up at the same time, and cells were collected at the end of the treatment, and the relative expression of PEDV N mRNA was detected using fluorescence quantitative PCR method.

### 2.8 Western blot assay

The proteins were extracted with RIPA lysate at a ratio of 1:100 (V/V), separated by 10% sodium dodecyl sulfate-polyacrylamide gel electrophoresis (SDS-PAGE), and transferred onto a PVDF membrane. After the transfer, the membrane was slightly cooled to room temperature, and the PVDF membrane was first washed with ultrapure water for a suitable period of time and then discarded, and then selected to add 5% BSA blocking solution and incubated at room temperature for 1 h, or 1× rapid blocking solution and incubated at room temperature for 20 min, respectively. the primary antibody was incubated overnight at 4°C. The primary antibody was incubated with TBST solution and then incubated with TBST solution. The primary antibody was incubated overnight at 4°C and was then washed three times with TBST solutio. Finally, the corresponding secondary antibody was added (1:1000) and incubated on a shaker at room temperature for 1 h. The membranes were then washed using TBST solution, and protein visualization was achieved by using the enhanced chemiluminescence (ELC) reagents. Target protein expression levels were analysed using ImageJ software (National Institutes of Health, Bethesda, MD, USA).

### 2.9 Network pharmacology

The relevant components of *S. stolonifera* were searched through the Herbal Etymology Review (HERB) platform(http://herb.ac.cn/), and eligible compounds were selected through the SWISSADME platform(http://www.swissadme.ch/) on the basis of the Linpinski 5 principle; The target proteins were searched in TCMSP (https://lilab-ecust.cn/pharmmapper/index.html) and PharmMapper (https://lilab-ecust.cn/pharmmapper/index.html) databases according to their composition, and the database information was summarized and duplicates were deleted. Filtering target information in the UniProt protein database (https://www.uniprot.org/). The GeneCards database(https://www.genecards.org/) was used to obtain the disease-related targets. After identifying the overlapping genes, protein-protein interactions were studied using the STRING protein interaction database(https://cn.string-db.org/) to exclude proteins that do not connect to other proteins, and the strength of the protein interactions was used as a basis for identifying core protein targets. GO and KEGG enrichment analyses of potential targets of *S. stolonifera* were performed and visualized using the Microbiotics online website(https://www.bioinformatics.com.cn/).

### 2.10 Co-Immunoprecipitation

The whole cell lysates were added lysis/rinsing buffer (containing 1% protease inhibitor mixture) and mix thoroughly, and were then centrifuged to collect the supernatant (4°C, 12,000 × g, 10 min) for the preparation of Input protein samples, in which the antibody was added and incubated overnight at 4°C to allow the formation of antigen-antibody complexes. Protein A/G magnetic bead suspensions were prepared in clean 1.5 mL centrifuge tubes by adding lysis/rinsing buffer and gently blowing to resuspend the beads. The tubes were then placed on a magnetic rack to ensure complete adsorption of the beads to the tube side, after which the supernatant was discarded. The antigen-antibody complexes were added to the pre-treated magnetic beads and incubate at 4°C for 4~6 h. After incubation, the magnetic beads were fully adsorbed and separated, and the supernatant was discarded. The remaining sample constituted the antigen-antibody-magnetic bead complex. The beads were resuspended in lysis/rinsing buffer, placed on the magnetic rack to adsorb to the side wall of the tube, and the supernatant was discarded. This wash step was repeated twice. To elute the proteins, an appropriate volume of PBS and 5× SDS-PAGE sample buffer was added to the washed complex, mixed well, and heated in a metal bath for 10 minutes. After cooling to room temperature, the tube was placed on a magnetic rack to adsorb the magnetic beads, and the supernatant was collected for Western blot analysis.

### 2.11 RT-qPCR

At the end of the cell treatment, the culture medium was discarded, and the total RNA was extracted according to the Accurate Biology Steady Pure Rapid RNA Extraction Kit, and the concentration was measured using the Nano Drop 2000. The extracted total RNA was reverse transcribed into cDNA using Accurate Biology Reverse Transcription Premix Kit. cDNA was obtained at the end of the reaction and can be stored at -20°C. The real-time fluorescence quantitative PCR was performed using a premixed qPCR kit from Accurate Biology. All primers for the test were commissioned to be synthesised by Sangon Biotech, as shown in **Table 1**.

**Table 1:** The primers used in RT-qPCR.

Species	Gene	(F: 5′-3′)	(R: 5′-3′)
PEDV	N	ACCAGTCCAAGAACAGAAACCAGTC	CATCCTTGACAGCAGCCACCAG
Chlorocebus	GAPDH	AGCCTCAAGATCATCAGCAATG	ATGGACTGTGGTCATGAGTCCTT
Porcine	GAPDH	AAGGAGTAAGAGCCCCTGGA	TCTGGGATGGAAACTGGAA
Chlorocebus	TNF-α	TCCAACCATGTGCTCCTCAC	TGGAGTCTCCCTCTGACAGG
	IL-1β	GCGGCAACGAGGATGACTT	TGGCTACAACAACTGACACGG
	IL-6	TGTGAAAGCAGCAAAGAG	AGTGTCCTCATTGAATCCA
	IL-8	CTGGCGGTGGCTCTCTTGG	TCTTTAGCACTCCTTGGCAAAACTG
	IL-10	CTGAGAACCACGACCCAGAC	AGGCATTCTTCACCTGCTCC
Porcine	TNF-α	TCGCCCACGTTGTAGCCAAT	TCCAGATAGTCGGGCAGGTT
	IL-1β	CCTTCAGTCCAGTCGCCTTCTCC	TGGCATCACCTTTGGCATCTTCTTC
	IL-6	AATCTGGGTTCAATCAGGAGACC	TCAGGTGCCCCAGCTACATT
	IL-8	AAATACGCATTCCACACCTTTCCAC	TGCTGTTGTTGTTGCTTCTCAGTTC
	IL-10	CTTCTCGCCTCCTCTCGTTGTC	AGTCTTCCAAGTGGTGCCTGTG

### 2.12 Molecular docking and visualization analysis

The 3D structure of the PEDV N protein was predicted using I-TASSER (https://zhanggroup.org/I-TASSER/), The corresponding small-molecule ligand was retrieved from the PubChem database (https://pubchem.ncbi.nlm.nih.gov/). The protein structure was preprocessed by adding hydrogen atoms and modifying amino acid residues using AutoDock Vina 1.2.0., The docking search space was defined using the Grid module, with the center coordinates set to (X=120, Y=134, Z=109) and the grid box size set to 165×135×108. A semi-flexible docking approach was employed, in which the ligand was flexible and the receptor was kept rigid. The docking conformation with the lowest binding energy was visualized and analyzed by PyMOL 2.3.0 and LigPlot 2.2.8 software. Sequence alignment analysis by using WeMol (https://wemol.wecomput.com/ui/#/frontend/home/navigator-board) (Katoh et al., 2005).

### 2.13 Statistical analysis

All experiments were repeated at least three times and the calculated results were presented as means ± standard error of mean (SEM). Data were analysed and processed using GraphPad Prism 9.0 and statistical analyses were performed using one-way ANOVA or student’s *t*-test. *p*<0.05 indicates a significant difference, *p*<0.01 indicates a highly significant difference and *p*>0.05 indicates no statistical significance.

## 3 Results

### 3.1 The inhibitory effects of *S. stolonifera* on PEDV replication across different cell types

**Figure 1:**
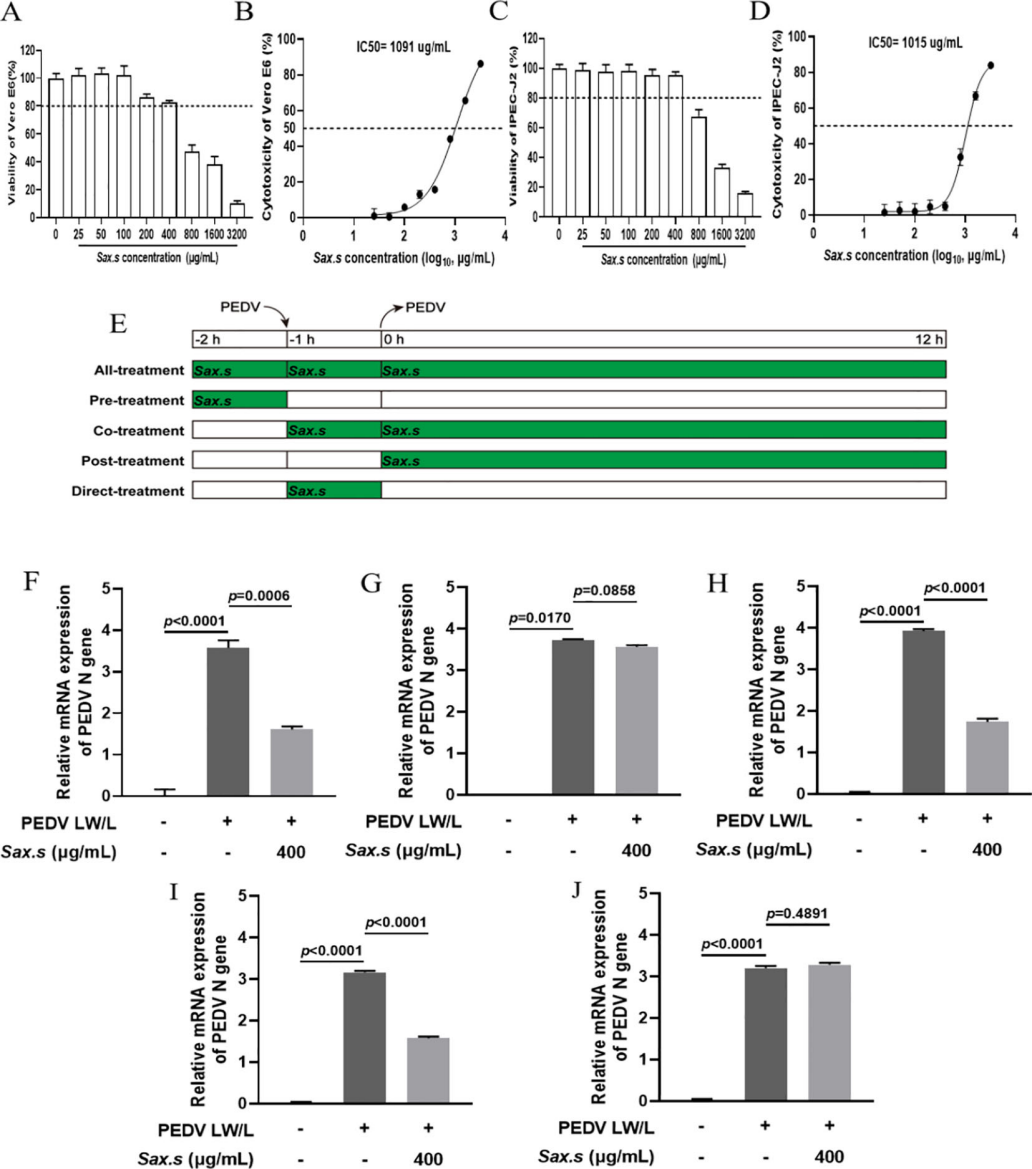
Inhibition of PEDV replication by *S. stolonifera*. **(A)** The cell viability of Vero E6 cells post Sax.s treatment. **(B)** The half maximal inhibitory concentration of *S. stolonifera* on Vero E6 cells. **(C)** The cell viability of IPEC-J2 cells post Sax.s treatment. **(D)** The half maximal inhibitory concentration of *S. stolonifera* on IPEC-J2 cells. **(E)** The diagram of different administration modes of Sax.s during PEDV infection. **(F)** The results of different *S. stolonifera* addition methods on PEDV resistance of all-treatment group. **(G)** pre-treatment group **(H)** co-treatment group **(I)** post-treatment group **(J)** direct-treatment group. The results are representative of three independent experiments (n=3). Data were represented as mean ± SEM. Student’s t test and one-way ANOVA with multiple comparisons was used for data analysis.

To evaluate the effects of *S. stolonifera* on PEDV infection, Vero E6 and IPEC-J2 cell lines were employed as *in vitro* models. IPEC-J2 cells, derived from the piglet small intestine, were chosen for their relevance to the primary infection sites, where the virus peaks, causing severe intestinal cell necrosis and detachment. Cytotoxicity assays showed a dose-dependent relationship between *S. stolonifera* concentration and cytotoxic effects on Vero E6 and IPEC-J2 cells after 12 h of treatment. Concentrations of *S. stolonifera* above 400 μg/mL significantly reduced cell viability, while treatments within the range of 0–400 μg/mL were well-tolerated. Both cell types maintained their morphological integrity and high viability, with Vero E6 cell viability consistently exceeding 80% (**Figure 1A**). The IC_50_ of *S. stolonifera* in Vero E6 cells was determined to be 1091 μg/mL (**Figure 1B**), highlighting the lack of significant cytotoxicity at lower concentrations. Similarly, IPEC-J2 cells showed no significant morphological changes at *S. stolonifera* concentrations below 400 μg/mL after 12 h of treatment, with cell viability consistently exceeding 80% (**Figure 1C**). The IC_50_ of *S. stolonifera* in IPEC-J2 cells was calculated as 1015 μg/mL (**Figure 1D**).

Several studies have demonstrated that various drug administration modes influence the antiviral efficacy of treatments (Chen et al., 2020; Liu et al., 2021). To determine the most effective method of *S. stolonifera* treatment, various administration modes were evaluated based on their antiviral efficacy (**Figure 1E**). The All-treatment, Co-treatment, and Post-treatment administration modes significantly reduced PEDV N mRNA expression levels (*p*<0.01), while Pre-treatment and Direct-treatment showed no significant inhibitory effects on PEDV (*p*>0.05) (**Figures 1F–J**). The results suggest that the Post-treatment mode has the strongest inhibitory effect on viral replication, demonstrating its efficacy in the shortest intervention period. Consequently, this administration mode was identified as optimal for managing PEDV infection in subsequent experiments.

**Figure 2:**
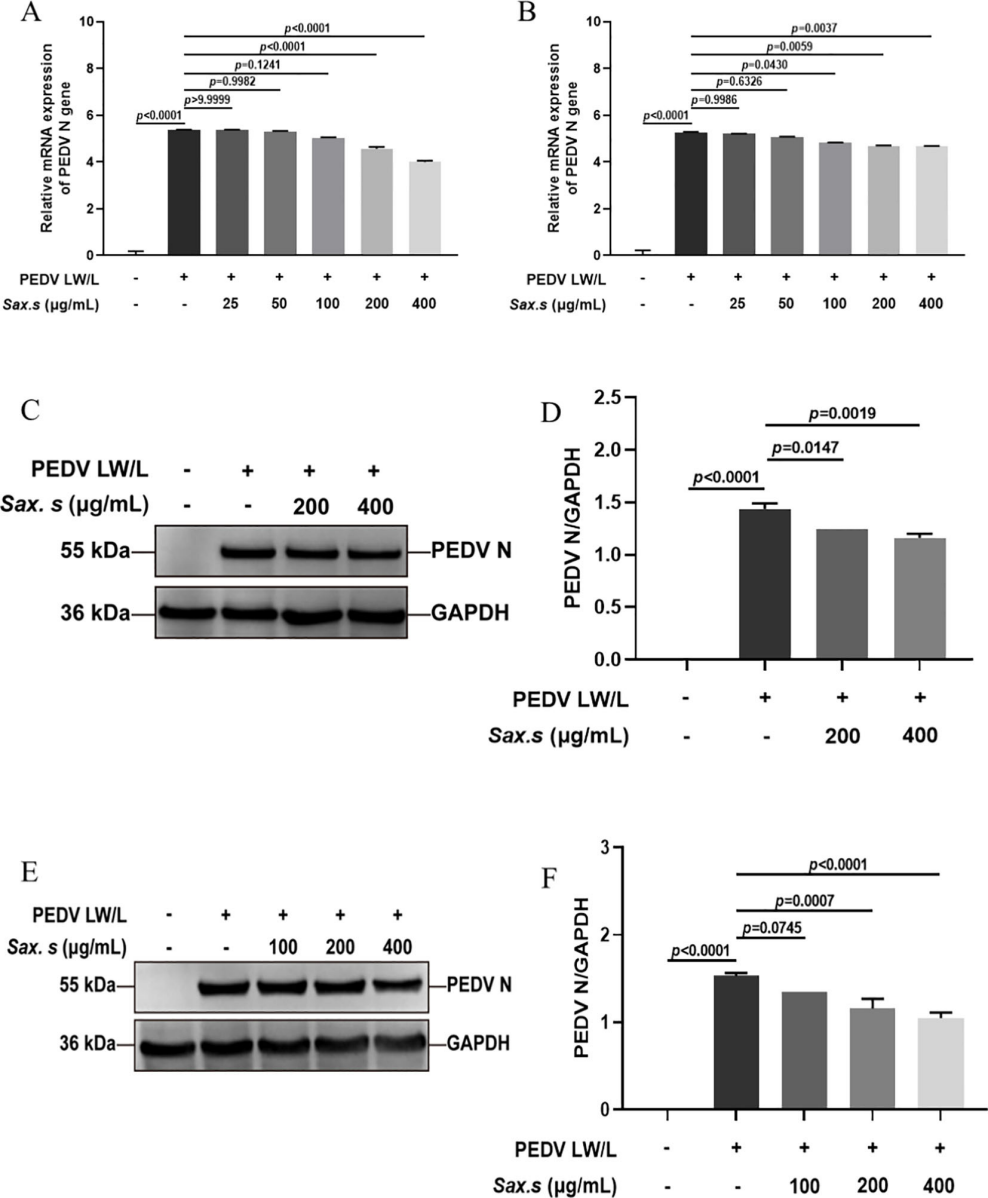
Different concentrations of *S. stolonifera* affect the expression levels of PEDV N gene. **(A)** The relative mRNA expression levels of of PEDV N gene with different concentrations of Sax.s in Vero E6 cells. **(B)** and IPEC-J2 cells. **(C, D)** The expression levels of PEDV N protein with different concentrations of Sax.s in Vero E6 cells. **(E, F)** The expression levels of PEDV N protein with different concentrations of Sax.s in IPEC-J2 cells. The results are representative of three independent experiments (n=3). Data were represented as mean ± SEM. Student’s t test and one-way ANOVA with multiple comparisons was used for data analysis.

To investigate the effects of varying *S. stolonifera* concentrations on PEDV N gene expression, *in vitro* assays were performed with different *S. stolonifera* concentrations applied to Vero E6 cells after a 12-hour exposure period. *S. stolonifera* significantly suppressed PEDV N gene expression at 200 and 400 μg/mL compared to the PEDV-infected control group (*p*<0.01) (**Figure 2A**). This suppressive effect was corroborated in IPEC-J2 cells, where *S. stolonifera* at 100, 200, and 400 μg/mL significantly reduced PEDV N gene expression (*p*<0.05) (**Figure 2B**). Collectively, these findings highlight that *S. stolonifera* at 200–400 μg/mL exhibits a potent *in vitro* inhibitory effect on PEDV, reducing PEDV N gene expression in a dose-dependent manner. To systematically evaluate the effect of *S. stolonifera* on PEDV N protein expression, comparative analyses were performed at varying concentrations. In Vero E6 cells, *S. stolonifera* at 200 and 400 μg/mL significantly reduced PEDV N protein expression compared to the PEDV-infected controls (*P*<0.05) (**Figures 2C, D**). This inhibitory effect was substantiated in IPEC-J2 cells (**Figures 2E, F**). In summary, *S. stolonifera* exerts a dose-dependent inhibitory effect on PEDV N protein expression *in vitro*, demonstrating significant antiviral activity.

### 3.2 The antiviral effects of *S. stolonifera* on distinct stages of PEDV infection

**Figure 3:**
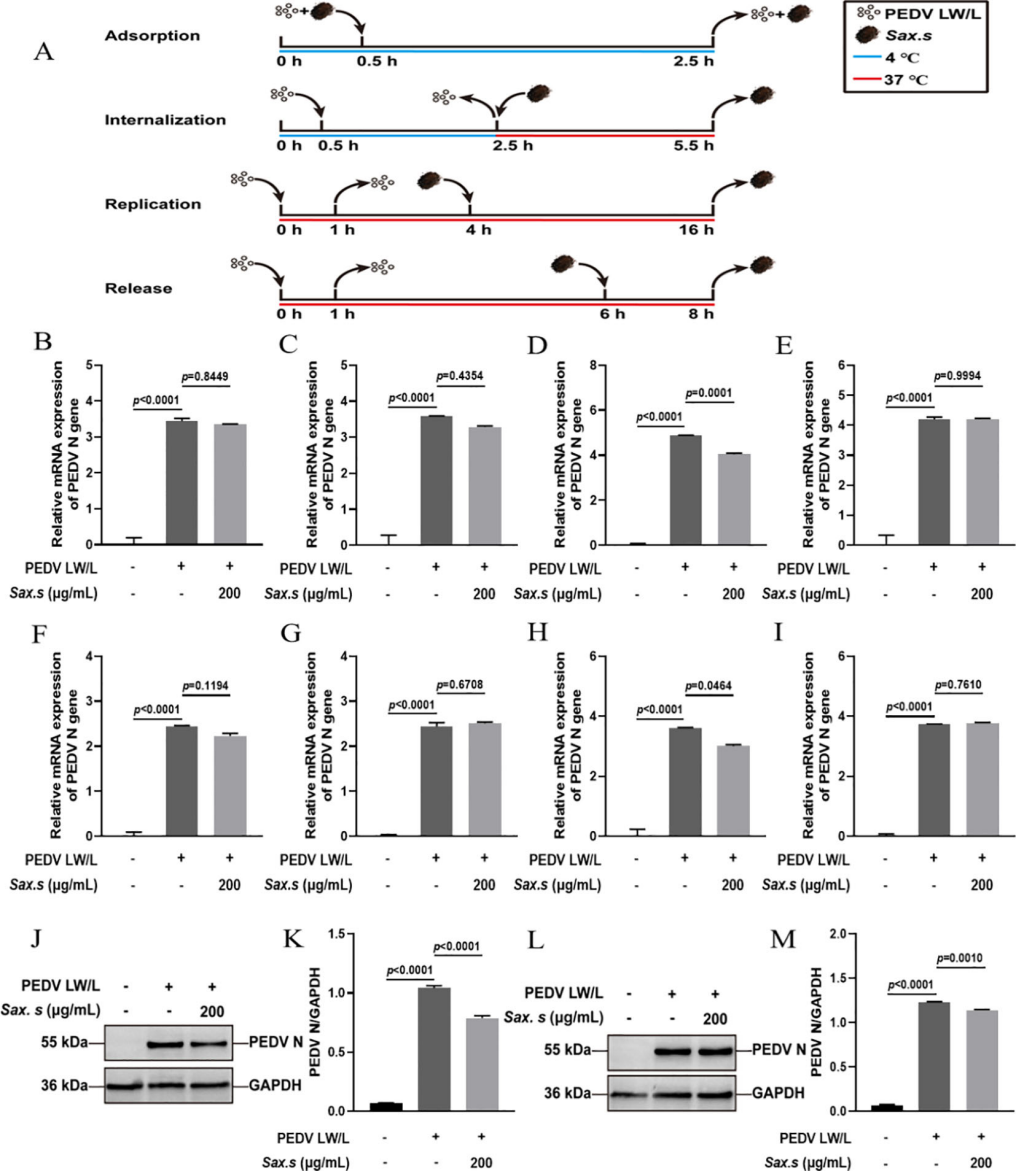
The antiviral effects of Sax.s on different infection steps of PEDV. **(A)** The schematic diagram of the effect of *S. stolonifera* on different PEDV infection stage. **(B)** The relative mRNA expression levels of N gene in Vero E6 cells by Sax.s in adsorption stage of PEDV. **(C)** internalization stage **(D)** replicative stage **(E)** release stage. **(F)** The relative mRNA expression levels of N gene in IPEC-J2 cells by Sax.s in adsorption stage of PEDV. **(G)** internalization stage **(H)** replicative stage **(I)** release stage. **(J, K)** The suppressive effects of *S. stolonifera* on the expressions PEDV N protein during the viral replication phase in Vero E6 cells. **(L, M)** IPEC-J2 cells. The results are representative of three independent experiments (n=3). Data were represented as mean ± SEM. Student’s t test and one-way ANOVA with multiple comparisons was used for data analysis.

To investigate the antiviral efficacy of *S. stolonifera* and its mechanism of action in the PEDV life cycle, we quantified viral N gene expression during key phases: adsorption, invasion, replication, and release (**Figure 3A**). Results revealed that 200 μg/mL *S. stolonifera* significantly inhibited the replication stage, reducing PEDV N gene mRNA expression in Vero E6 cells (*p*<0.01) (**Figures 3B–E**). However, this concentration showed no significant antiviral activity during adsorption, invasion, or release stages. Similar results were found in IPEC-J2 cells, where 200 μg/mL *S. stolonifera* significantly suppressed PEDV replication (*p*<0.05) but had negligible effects on other viral life cycle stages (**Figures 3F–I**). Next, we examined the effect of *S. stolonifera* on N protein expression during PEDV replication. Data showed that 200 μg/mL *S. stolonifera* significantly reduced PEDV N protein expression in Vero E6 cells (*p*<0.0001) (**Figures 3J, K**). This inhibitory effect was also observed in IPEC-J2 cells, where 200 μg/mL *S. stolonifera* significantly reduced PEDV N protein expression during replication (*p*=0.0010) (**Figures 3L, M**).

### 3.3 Evaluation of the anti-inflammatory properties of *S. stolonifera* in PEDV-specific cell lines

**Figure 4:**
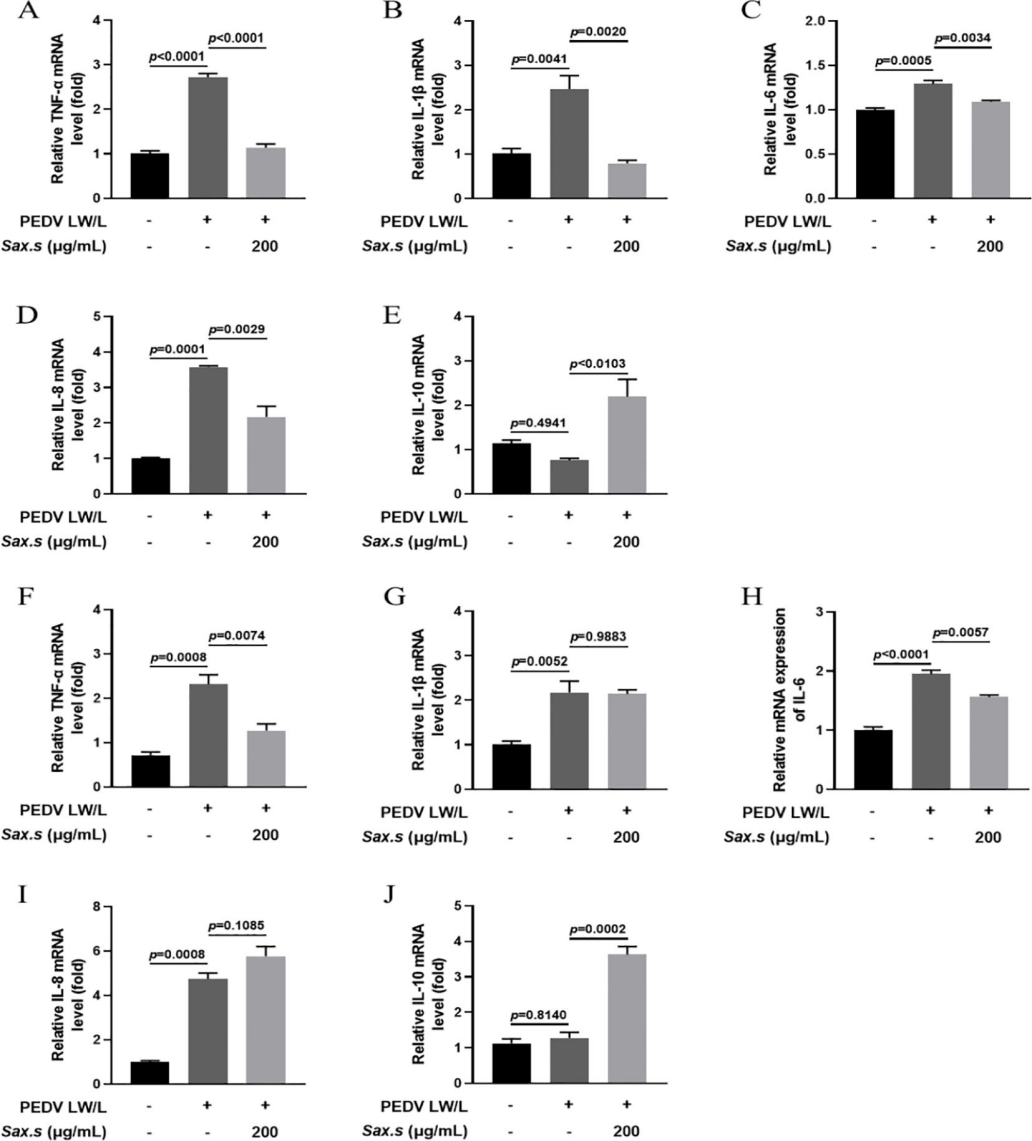
Assessing the anti-inflammatory impacts of *S. stolonifera* on PEDV-specific cell lines. **(A)** The relative mRNA expression levels of TNF-α. **(B)** IL-1β **(C)** IL-6 **(D)** IL-8 **(E)** IL-10 in Vero E6 cells. **(F)** The relative mRNA expression levels of TNF-α. **(G)** IL-1β **(H)** IL-6 **(I)** IL-8 **(J)** IL-10 in IPEC-J2 cells. The results are representative of three independent experiments (n=3). Data were represented as mean ± SEM. Student’s t test and one-way ANOVA with multiple comparisons was used for data analysis.

Viral replication relies on host cells as the main energy source. Infected cells, in turn, activate the host’s innate immune system through a cascade of responses. This activation alters the expression of inflammatory cytokines and chemokines (Nguyen et al., 2021). Research shows that PEDV infection induces distinct cytokine profiles in host cells. Identifying the specific cytokines and chemokines involved is crucial for understanding the host’s immune response to viral infection (Huang et al., 2024). To investigate *S. stolonifera*’s antiviral mechanism, we used Real-time PCR to measure the transcriptional levels of key inflammatory cytokines— (TNF-α, IL-1β, IL-6, IL-8, and IL-10)—in PEDV-infected cells after *S. stolonifera* treatment. Results showed significant upregulation of TNF-α, IL-1β, IL-6, and IL-8 mRNA levels in Vero E6 cells post-infection. Notably, *S. stolonifera* at 200 μg/mL significantly reduced this upregulation (*p*<0.01) (**Figures 4A–D**). Moreover, the same concentration of *S. stolonifera* significantly increased the expression level of IL-10 mRNA (**Figure 4E**). Validation in IPEC-J2 cells confirmed the upregulation of TNF-α, IL-1β, IL-6, and IL-8 mRNA levels following PEDV infection. *S. stolonifera* at 200 μg/mL significantly reduced TNF-α and IL-6 mRNA levels but had no significant effect on IL-1β and IL-8 expression (**Figures 4F–I**). Additionally, *S. stolonifera* significantly upregulated IL-10 expression, highlighting its immunomodulatory potential (**Figure 4J**). These results suggest that PEDV induces pro-inflammatory cytokine expression, while *S. stolonifera* modulates inflammation by reducing pro-inflammatory factors and increasing IL-10 levels, demonstrating its potential anti-inflammatory effects.

### 3.4 Network pharmacology analysis predicts that *S. stolonifera* mitigates PEDV infection through modulation of the p53 signaling pathway

**Figure 5:**
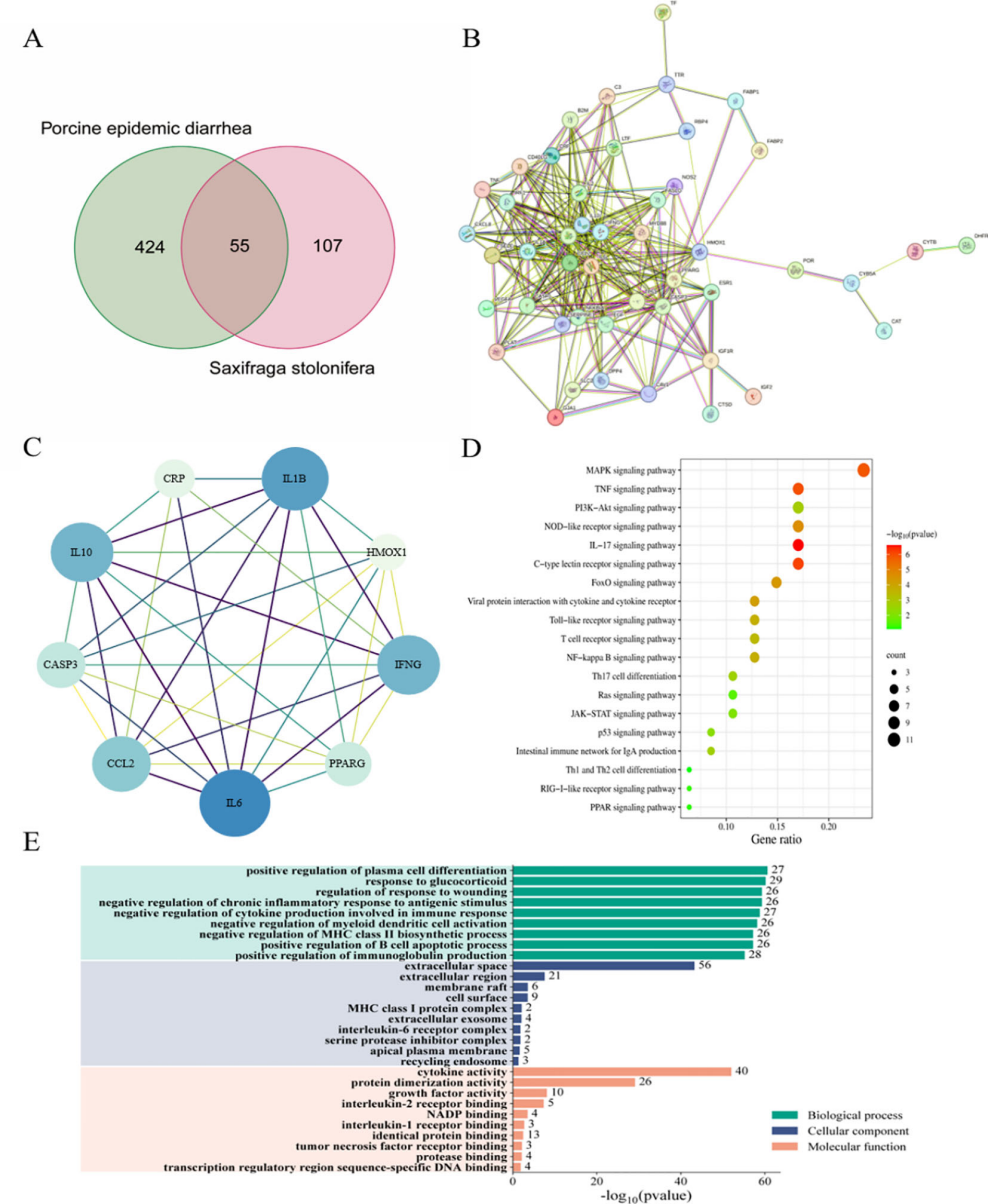
Network pharmacology predicts the mechanism of anti-pedv action of *S. stolonifera*. 55 overlapped target genes were from three databases (TCMSP, PharmMapper and GeneCards). **(A)** 55 overlapped target genes were used to constructed PPI network and hub genes in PPI network. **(B, C)** 55 overlapped genes was analysis by KEGG, which enriched in 20 pathways. The darker the color, the greater the weight, and the larger the circle, the greater the genenumber. **(D)** GO annotations were analysed for 55 overlapping genes. **(E)** BP/CC/MF are all shown in the figure, and the specific meanings can be found in the text on the left side of the bar graphs.

The shared targets between the drug components and disease were depicted using Venn diagrams, identifying 55 common targets, which were subsequently imported into the String database (**Figure 5A**). Cytoscape software was utilized to visualize the protein-protein interaction (PPI) network of *S. stolonifera* and identify the direct and indirect regulation of the targets (**Figure 5B**). Using the Cytoscape plugin CentiScaPe 2.2, core targets including IL10, CCL2, IL6, IFNG, IL1B, CRP, CASP3, HMOX1, and PPARG were identified based on Betweenness unDir, Closeness unDir, and Degree unDir metrics (**Figure 5C**). The corresponding components of these targets were deduced as focal gallic acid, quercetin, coumarin, caffeic acid, arbutin, succinic acid, 6,7-dihydroxycoumarin, gallic acid, and hydroquinone. GO and KEGG pathway enrichment analyses revealed that *S. stolonifera* combats PEDV infection via the MAPK, TNF, PI3K-Akt, JAK-STAT, and p53 signaling pathways (**Figures 5D, E**).

### 3.5 *S. stolonifera* treatment disrupted the interaction between PEDV N protein and p53 protein

**Figure 6:**
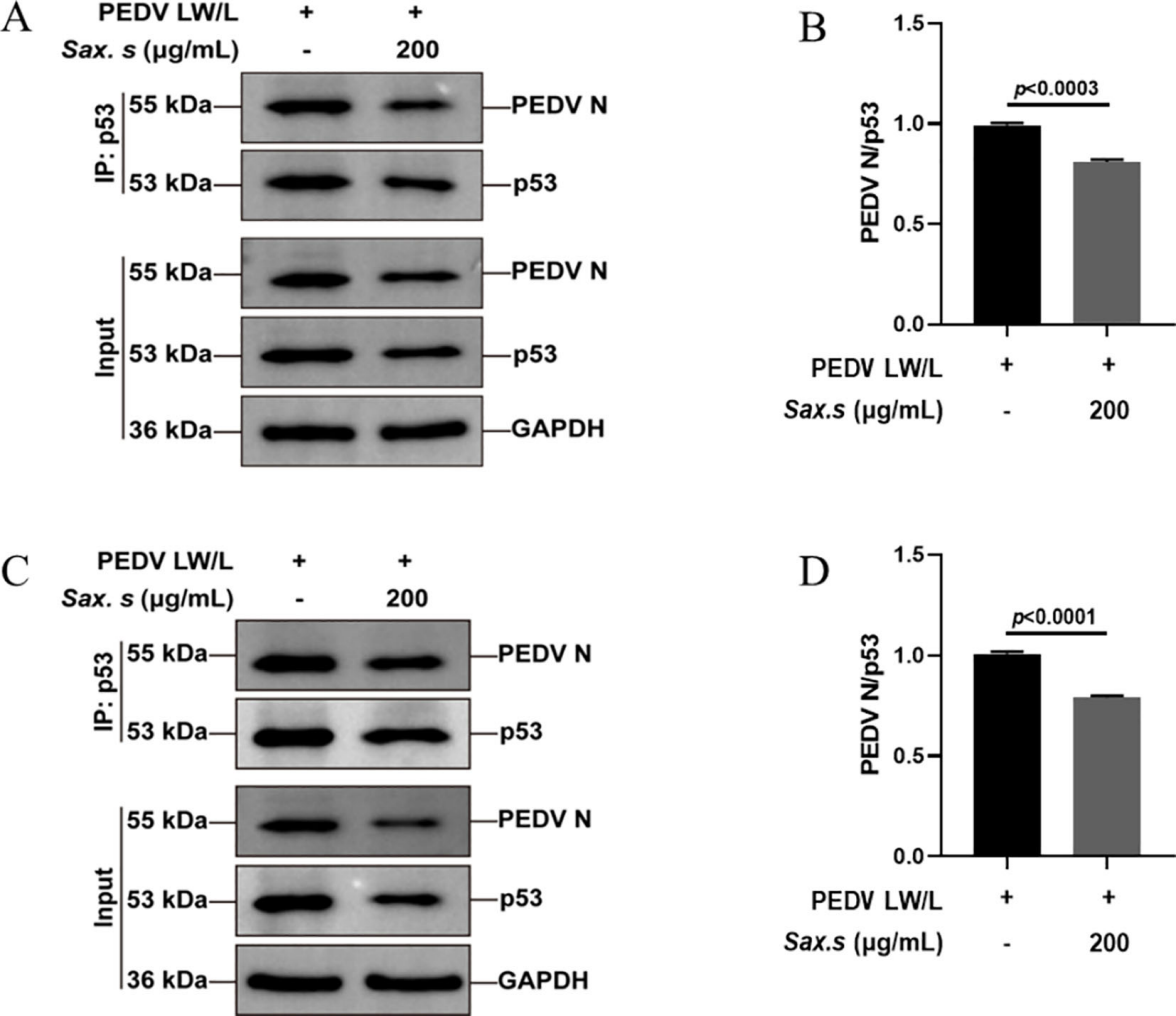
*S. stolonifera* treatment disrupts PEDV N protein-p53 protein interactions. **(A, B)** Anti-p53 antibody based co-IP analysis of the interaction between PEDV N protein and p53 in the Vero E6 cells post the treatment of *S. stolonifera*; **(C, D)** Anti-p53 antibody based co-IP analysis of the interaction between PEDV N protein and p53 in the IPEC-J2 cells post the treatment of *S. stolonifera*. The results are representative of three independent experiments (n=3). Data were represented as mean ± SEM. Student’s t test and one-way ANOVA with multiple comparisons was used for data analysis.

Previous studies have shown that the binding of the PEDV N protein to p53 sustains elevated nuclear p53 levels, mediating S-phase arrest through activation of the p53-DREAM pathway (Su et al., 2021). This interaction highlights a potential antiviral strategy targeting the host-virus interface. Using a Co-Immunoprecipitation (Co-IP) assay, we explored the effect of *S. stolonifera* on the molecular interaction between the PEDV N protein and the tumor suppressor p53 in Vero E6 cells. We found that 200 μg/mL *S. stolonifera* significantly reduced the formation of the complex between the PEDV N protein and p53 (*p*<0.0003) (**Figures 6A, B**). Moreover, this treatment effectively inhibited PEDV replication, as shown by comparison with the PEDV-infected control group, highlighting the antiviral potential of *S. stolonifera*. The inhibitory effect of *S. stolonifera* on the interaction between the PEDV N protein and p53 was further confirmed in IPEC-J2 cells (**Figures 6C, D**). These results demonstrate the broad applicability of the antiviral mechanism of *S. stolonifera*, emphasizing its potential as a therapeutic option for various cell types affected by PEDV infection.

### 3.6 *S. stolonifera* exhibited antiviral effects primarily by disrupting the viral activation of the p53-DREAM signaling pathway

**Figure 7:**
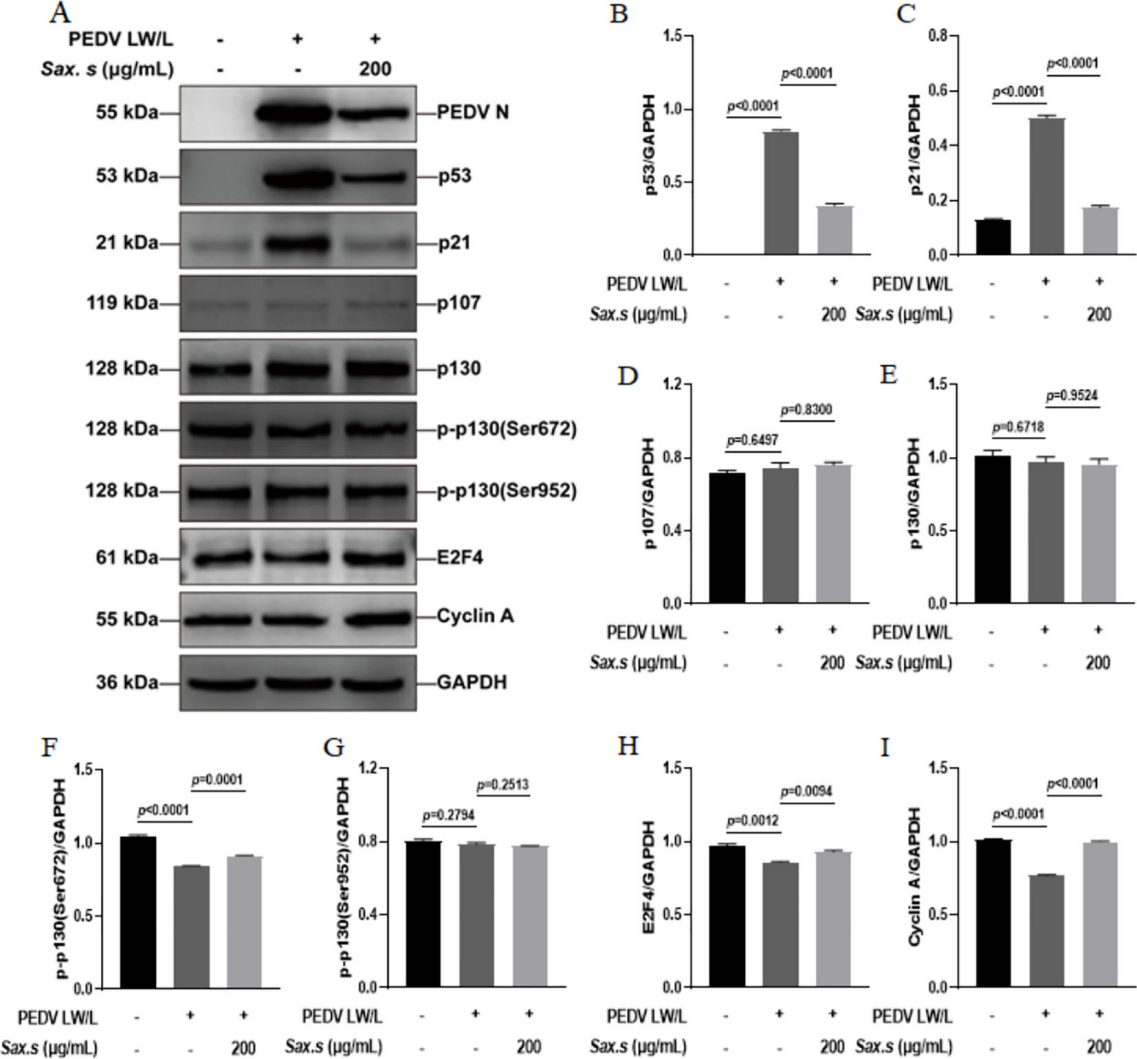
*S. stolonifera* exerts antiviral effects by interfering with the p53-dream signaling pathway in Vero E6 cells. **(A)** Analysis of the expression levels of p53-DREAM signalling pathway-related proteins in Vero E6 cells. **(B)** The relative expression levels of p53. **(C)** p21 **(D)** p107 **(E)** p130 **(F)** p130 phosphorylated (Ser672) **(G)** p130 phosphorylated (Ser952) **(H)** E2F4 **(I)** CyclinA were analyzed and were shown in the graphs. The results are representative of three independent experiments (n=3). Data were represented as mean ± SEM. Student’s t test and one-way ANOVA with multiple comparisons was used for data analysis.

**Figure 8:**
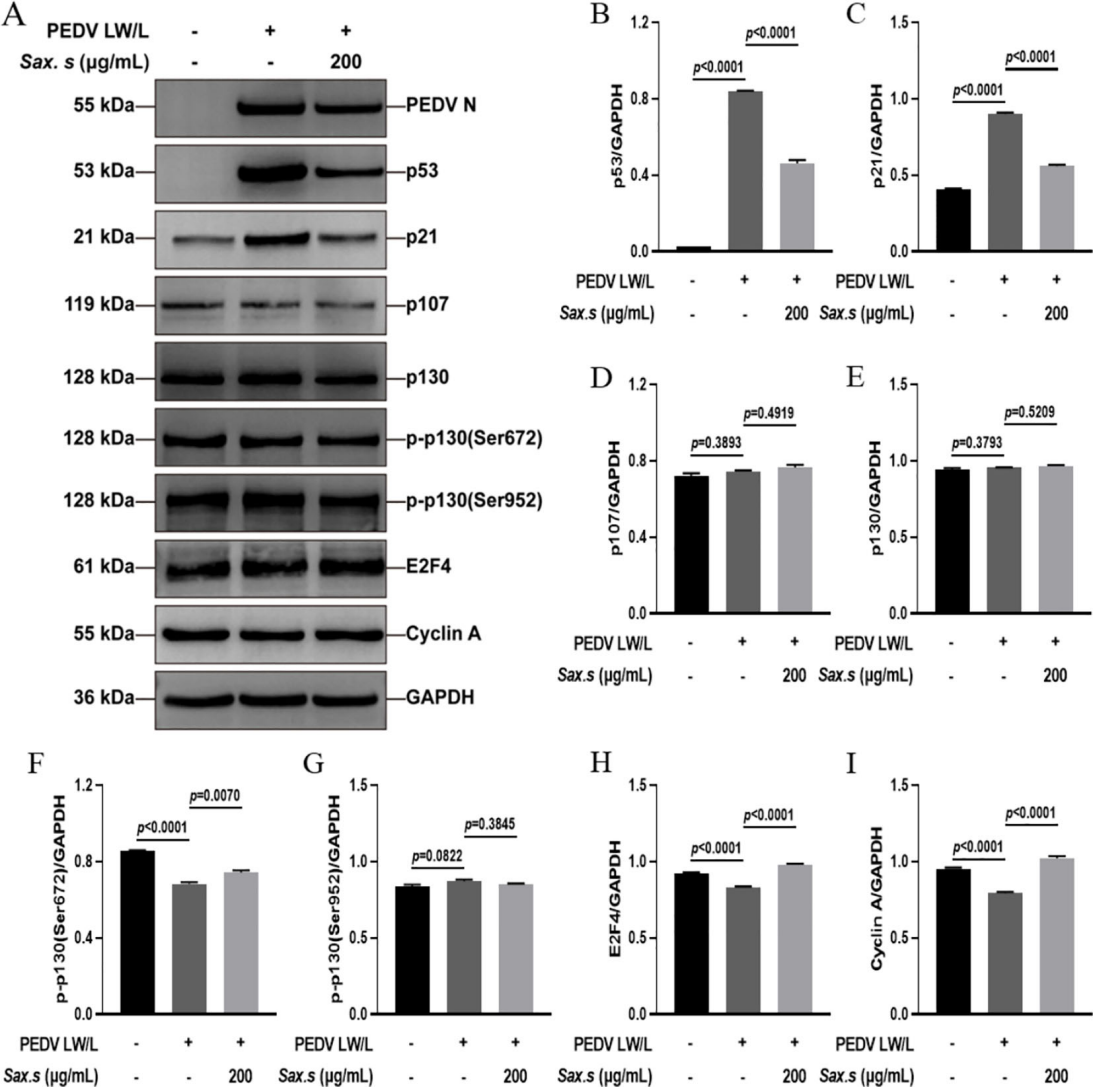
*S. stolonifera* exerts antiviral effects by interfering with the p53-dream signaling pathway in IPEC-J2 cells. **(A)** Analysis of the expression levels of p53-DREAM signalling pathway-related proteins in IPEC-J2 cells. **(B)** The relative expression levels of p53. **(C)** p21 **(D)** p107 **(E)** p130 **(F)** p130 phosphorylated (Ser672) **(G)** p130 phosphorylated (Ser952) **(H)** E2F4 **(I)** CyclinA were analyzed and were shown in the graphs. The results are representative of three independent experiments (n=3). Data were represented as mean ± SEM. Student’s t test and one-way ANOVA with multiple comparisons was used for data analysis.

We conducted a thorough evaluation of *S. stolonifera* effects on the expression dynamics of proteins involved in the p53-DREAM signaling cascade using Western blot analysis (**Figure 7A**). Our analysis revealed a marked upregulation of p53 and its downstream effector p21 upon PEDV infection (**Figures 7B, C**). We also observed a significant downregulation in the levels of phosphorylated p130 at Ser672, E2F4, and Cyclin A following infection (**Figures 7F, H, I**). In contrast, the expression of p107, total p130, and p130 phosphorylated at Ser952 remained unchanged after PEDV infection (*p*>0.05) (**Figures 7D, E, G**). Interestingly, treatment with 200 μg/mL *S. stolonifera* significantly reduced the expression levels of p53 and p21 while robustly upregulating phosphorylated p130 (Ser672), E2F4, and Cyclin A, suggesting a potent regulatory effect on the p53-DREAM signaling pathway. These effects of *S. stolonifera* were also characterized by downregulation of p53 and p21 in IPEC-J2 cells (**Figures 8A–C**), alongside upregulation of phosphorylated p130 (Ser672), E2F4, and Cyclin A (**Figures 8F, H, I**). These coordinated actions suggest a complex mechanism by which *S. stolonifera* may suppress viral replication via the p53-DREAM signaling pathway.

### 3.7 The active ingredients of *S. stolonifera* bind to the PEDV N protein, inducing antiviral effects

**Figure 9:**
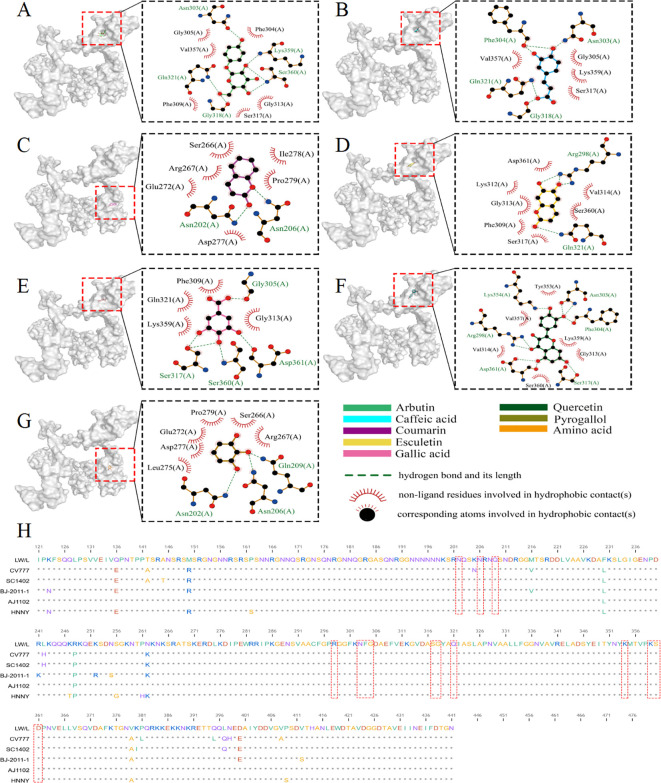
Core ingredient of *S. stolonifera* attaches to the PEDV N protein to inhibit PEDV infection. **(A)** The amino acids of the N protein bind to arbutin through hydrogen bonds and hydrophobic forces. **(B)** caffeic acid **(C)** coumarin **(D)** esculetin **(E)** gallic acid **(F)** quercetin **(G)** pyrogallol **(H)** The sequence alignment of N gene.

The PEDV N protein is abundantly expressed during the early stages of viral infection, and pigs produce high levels of anti-N protein antibodies, which are highly conserved across different PEDV strains (Ma et al., 2019). Consequently, the N protein serves as a valuable tool for exploring the mechanisms by which host antiviral genes inhibit PEDV replication, offering new insights for PEDV prevention and control. Molecular docking was employed to investigate the binding sites of core *S. stolonifera* components with the N protein. The results were visualized using Pymol, with smaller free binding energy indicating a more stable bound conformation and a higher likelihood of interaction. Binding activity was considered favorable when the energy was ≤-5.0 kcal-mol^-1^. The analysis revealed that arbutin, quercetin, caffeic acid, coumarin, gallic acid, pyrogallol, and esculetin exhibited strong binding activity with the N protein. Additionally, the amino acid residues at the docking sites were visualized using Ligplot software (**Figures 9A–G**). Five N gene sequences were selected for comparison (Wang et al., 2021; Guo et al., 2024). The CV777 and SC1402 strains belong to genotype GI, while the other three strains are classified as epidemic or virulent strains of genotype GII. We found that the amino acids involved in hydrogen bonding at the docking site between *S. stolonifera* and the N protein are conserved across these sequences (**Figure 9H**). These findings suggest that *S. stolonifera* may bind to conserved amino acids via hydrogen bonding with the N protein, thereby inhibiting infection.

## 4 Disscussion

Similar to other coronaviruses, PEDV is prone to mutations that may enable immune evasion, potentially reducing vaccine efficacy. Current epidemiological data indicate that PEDV is the most prevalent and lethal Class II animal disease in China. Although pharmaceuticals and vaccines are available, they have been insufficient to curb the spread of PEDV outbreaks (Jiao et al., 2021). Considering the substantial impact of PEDV on piglets and the pig-breeding industry, developing effective antiviral drugs for PEDV infections is urgently needed.Coronavirus nucleocapsid proteins are abundantly expressed in infected cells and play a key role in promoting viral replication and evading the host immune response. Studies have identified nucleocapsid proteins as promising targets for anticoronavirus drug therapy, with evidence indicating that the PEDV N protein induces host cell-cycle arrest and prolongs the S-phase via interaction with p53 (Xu et al., 2013; Su et al., 2021; Zhu et al., 2022).

Chinese herbal medicines, abundant and widely distributed across China, provide valuable resources for drug development. These herbs are characterized by their multi-component, multi-target, and low-residue profiles, making them focal points for antiviral research and widely used in clinical practice. Cho et al. screened 333 aqueous extracts of Chinese herbal medicines for antiviral activity against PEDV, identifying Korean horny goatweed and honeysuckle as particularly effective (Cho et al., 2012). The aqueous extract of Korean horny goatweed, known for its antitoxic properties, has been used as a feed additive for piglets, effectively alleviating intestinal lesions, improving fecal consistency, and reducing viral loads in PEDV-infected animals. Xu et al. found that Aloe vera extract directly inactivates PEDV particles and inhibits viral replication, with a dosage of 100 mg/kg effectively protecting infected piglets (Xu et al., 2020). Cao et al. reported that Moringa oleifera leaf extract modulates reactive oxygen species and inflammatory responses induced by PEDV, inhibiting viral replication through effects on the apoptosis pathway (Cao et al., 2022). Trinh et al. evaluated the anti-PEDV potential of aqueous and ethanolic plant extracts, identifying the ethanolic extract of Garcinia cambogia as the most potent antiviral agent (Trinh et al., 2021). Compared to other natural antiviral agents, *S. stolonifera* in this study exhibits a dual mechanism by directly targeting the viral N protein and modulating the p53-DREAM signaling pathway. Moreover, the multi-component, multi-target, and multi-pathway synergistic effects inherent to traditional Chinese medicine enhance viral control and reduce the risk of drug resistance. Herbal medicines also offer several additional advantages in treating PED, including high safety, minimal side effects, and environmental sustainability. Network pharmacology, an emerging field, focuses on addressing the causal mechanisms of diseases rather than simply alleviating symptoms (Nogales et al., 2022). To understand how *S. stolonifera* influences viral replication, we performed network pharmacology analysis to identify key genes shared between *S. stolonifera* and PEDV. Through Gene Ontology (GO) and Kyoto Encyclopedia of Genes and Genomes (KEGG) pathway analyses, we identified several key signaling pathways associated with these genes, including the MAPK, PI3K-Akt, NF-κB, and p53 signaling pathways. These pathways are crucial in cell cycle regulation and antiviral immune responses, providing deeper insights into the therapeutic potential of *S. stolonifera* against PEDV.

This study found that *S. stolonifera* primarily impacts the viral replication stage. Viruses manipulate the cell cycle through distinct molecular pathways, inducing phase-specific arrest to create a cellular environment favorable for replication (Quan et al., 2023). This strategy is commonly used by viruses to exploit host cell resources for replication. A thorough understanding of how viruses modulate the host cell cycle is crucial for developing effective antiviral therapeutics. Studies have shown that PEDV infection induces G0/G1 phase arrest in Vero cells by downregulating Cyclin E expression, which can be alleviated by inhibiting the p53 signaling pathway (Xu et al., 2013; Xu et al., 2015; Sun et al., 2018). The PEDV M and N proteins reduce Cyclin A expression, causing S-phase arrest in intestinal epithelial cells (IECs). The DNA damage response pathway regulates checkpoint controls at the G1, S, and G2 phases, playing a key role in DNA repair and apoptosis regulation (Xu et al., 2015). This aligns with earlier findings that genomic damage-induced cell cycle arrest activates p53 protein, further inducing apoptosis (Vidya Priyadarsini et al., 2010). Moreover, the PEDV N protein interacts with p53, promoting its ubiquitination and modulating the NF-κB pathway by downregulating p65 and p50 expression. This interaction is essential for viral replication. Hyperoside, a compound derived from hawthorn, alleviates S-phase arrest caused by the PEDV N protein by disrupting its interaction with p53, thereby inhibiting PEDV replication *in vitro (* Su et al., 2021). This study used Vero E6 and IPEC-J2 cell lines to examine the antiviral activity of *S. stolonifera* and its effects on the p53-DREAM signaling pathway. *S. stolonifera* was found to inhibit the interaction between the PEDV N protein and p53, exerting antiviral effects by modulating p53 and p21 expression, enhancing p130 phosphorylation, and upregulating E2F4 and Cyclin A, and this is consistent with previous reports. Small molecules commonly interact with protein targets to induce biological activities and pharmacological effects. Among the compounds in *S. stolonifera*, 6,7-dihydroxycoumarin, quercetin, caffeic acid, coumarin, and arbutin are identified as the primary bioactive constituents (Wang et al., 2018). Oral quercetin supplementation has been shown to enhance COVID-19 recovery by inhibiting ACE2 and TMPRSS2 expression (Houghton et al., 2024). Caffeic acid is well-known for its diverse therapeutic properties, including anti-inflammatory, antiviral, anticancer, and neuroprotective effects, and is often included in formulations to harness these benefits (Goyal et al., 2025). Esculetin and coumarin, both common in traditional Chinese medicine, have shown significant potential in inhibiting cancer cell proliferation and inducing cell cycle arrest (Liu et al., 2024). Arbutin, another key component, is recognized for its anti-inflammatory and antioxidant properties. These properties are essential for reducing oxidative stress and neuroinflammation, contributing to cellular health and tissue integrity (Sharma et al., 2024).

To fully harness the therapeutic potential of *S. stolonifera*, a comprehensive and systematic approach is essential. This begins with an in-depth investigation into the pharmacological mechanisms underpinning its exogenous and endogenous effects. Additionally, optimizing dosage forms is critical to enhance the bioavailability and therapeutic index of *Saxifrage*’s active constituents. Such optimization involves the development of advanced pharmaceutical formulations designed to ensure the safe and effective delivery of its bioactive compounds. Equally important is the establishment of evidence-based application guidelines. These guidelines should be firmly rooted in robust scientific research and validated through rigorous clinical trials, providing precise recommendations for the use of *S. stolonifera* in managing porcine epidemic diarrhea. *S. stolonifera* shows promise as a standalone therapeutic agent or in combination with complementary treatments, potentially enhancing its efficacy in clinical applications. To unlock its full potential, it is imperative to deepen our scientific understanding of *S. stolonifera*’s pharmacological properties. This requires further research to elucidate its mechanisms of action, as well as comprehensive evaluations of its safety and efficacy. By integrating these findings with the principles of traditional Chinese medicine (TCM) diagnosis and treatment, *S. stolonifera* can be applied responsibly and effectively in clinical practice.

In summary, our study demonstrates that *S. stolonifera* inhibits PEDV infection by interacting with both the virus and host cells. Firstly, computational docking results indicate a potential interaction between Sax. s and the viral N protein, which may contribute to the inhibition of viral replication and disruption of virus assembly and release. Secondly, *S. stolonifera* promotes recovery from cell cycle arrest in PEDV-infected cells through modulation of the p53-DREAM signaling pathway. These findings contribute to a deeper understanding of *S. stolonifera* protective role against PEDV and offer valuable insights for the development of antiviral therapeutics. They also suggest innovative strategies for ‘target-driven’ pharmaceutical research, especially in the context of traditional Chinese medicine. However, it should be noted that these conclusions are based on *in vitro* models, which cannot fully replicate the complex physiological conditions of living organisms. Therefore, the clinical efficacy and pharmacodynamic relevance of *S. stolonifera* require further *in vivo* validation. Verifying the antiviral activity and elucidating the mechanisms of its active constituents against PEDV will be the focus of our future studies.

## Data Availability

The raw data supporting the conclusions of this article will be made available by the authors, without undue reservation.
